# Functional outcomes of proximal tibia fractures in adults using KOOS

**DOI:** 10.6026/973206300222667

**Published:** 2026-04-30

**Authors:** Amol Kawde, Kartikeya Singh, Sanjul Bansal, Abhishek Keshav, Chayan Jain, Pratima Nagale, Shrajay Singh Rawat, Dharmendra Verma1, Sachin Parmar

**Affiliations:** 1 Department of Orthopaedics, AtalBihari Vajpayee Government Medical College (ABVGMC), Vidisha, Madhya Pradesh India; 2 Department of Orthopaedics, LNCT Medical College and Sevakunj Hospital, Indore, Madhya Pradesh, India; 3 Department of Orthopaedics, AIIMS, Bhopal, Madhya Pradesh, India; 4 Department of Orthopaedics, SAMC and PGI, Indore Madhya Pradesh India; 5 Department of Community Medicine, V.K.S. Government Medical College, Neemuch, Madhya Pradesh, India

**Keywords:** Proximal tibia fracture, schatzker's fracture classification, knee injury and osteoarthritis outcome Score (KOOS)

## Abstract

Complex intra-articular injuries like proximal tibial fractures need validated patient-reported functional outcomes. Functional outcomes of 30 adults undergoing locking plate ORIF for closed proximal tibia fractures were assessed via KOOS at 1.5, 3 and 6 months postoperatively (September 2022-February 2024). The cohort consisted of middle-aged males (76.7%, mean age 44.33 ± 13.19 years) with the most common fracture patterns being Schatzker Type-5 (46.7%) and Type-6 (30%). Patients experienced bone union at 15.03 ± 1.61 weeks without complications. Overall KOOS scores improved significantly from 6 weeks to 6 months (51.80 ± 8.79 to 85.73 ± 4.65, p<0.001), with sport/recreation function gaining the most (30.83 to 74.50). Open reduction and internal fixation with locking plate constructs improves functional outcomes in proximal tibial fractures, and KOOS can assess mid-term functional recovery across Schatzker classification types.

## Background:

Proximal tibial fractures represent a significant challenge in orthopedic trauma, accounting for approximately 1% of all fractures with an annual incidence estimated at 10 per 100,000 inhabitants [1]. These injuries predominantly affect individuals between the ages of 40 and 60 years, with a higher prevalence in men, although elderly women are more susceptible to low-energy fractures [2]. The mechanism of injury varies considerably, with high-energy trauma such as motor vehicle accidents being the primary cause in younger males, while low-energy mechanisms including simple falls predominate in elderly patients [3]. Anatomical reduction and mechanical axis restoration are needed for optimal knee joint function after fractured articular surfaces. Diagnostic imaging and surgery help treat proximal tibial fractures. Conservative ORIF management includes locked plating, intramedullary nailing and arthroscopic reduction [4]. Despite these advances, complications remain substantial, with reported rates ranging from 19% to 30%, including knee stiffness, infection, malunion, nonunion and post-traumatic osteoarthritis [5, 6]. The complexity of these fractures, combined with potential soft tissue damage and neurovascular injuries, necessitates comprehensive preoperative planning and meticulous surgical execution. Assessment of functional outcomes following proximal tibial fracture treatment is paramount for evaluating the effectiveness of therapeutic interventions and guiding clinical decision-making [7]. Traditional outcome measures have included radiological assessment using modified Rasmussen criteria and various functional scoring systems [8]. However, patient-reported outcome measures (PROMs) provide valuable insights into the patient's perspective regarding pain, symptoms, daily activities and quality of life, which may not be fully captured by clinical and radiological assessments alone [9]. The KOOS self-report knee injury/OA questionnaire has five subscales: Pain, Symptoms, ADL, Sport/Rec and QOL [10]. Recent validation shows that it works for lateral tibial plateau fractures, with strong test-retest reliability (ICC 0.6-0.9) and a high responsiveness at 6 and 12 months [11]. The KOOS tracks both surgical and non-surgical recovery by measuring pain relief and functional gains more accurately than generic measures [12]. Because proximal tibial fractures can make it hard to move around, the KOOS gives a standard, evidence-based way to look at and compare treatment results [13]. Therefore, it is of interest to assess the functional outcomes of proximal tibia fractures across all Schatzker classification types using the validated Knee injury and Osteoarthritis Outcome Score (KOOS).

## Methodology:

This cross-sectional observational study was conducted in the Department of Orthopaedic at Sri Aurobindo Medical College and Postgraduate Institute, Indore, Central India, from September 2022 to February 2024. The study protocol received institutional ethics committee approval prior to patient enrollment. The study population comprised 30 adult patients diagnosed with proximal tibia fractures attending the orthopedic outpatient department and emergency casualty services. All Schatzker classification types (Type I through Type VI) were included to provide comprehensive representation of fracture patterns. The Schatzker classification system categorizes tibial plateau fractures based on fracture morphology and serves as a prognostic indicator for functional outcomes. Inclusion criteria encompassed patients aged 18 years or above presenting with closed proximal tibia fractures. Exclusion criteria eliminated patients with open fractures, concurrent ipsilateral lower limb fractures, associated neurovascular injuries, or those who declined informed consent. All eligible patients received detailed explanation about the study in their native language and voluntary written informed consent was obtained from either the patient or their legally acceptable representative. Comprehensive physical and clinical examinations were performed with standard anteroposterior and lateral knee radiographs obtained for fracture characterization. Initial management comprised posterior above- knee splintage, limb elevation and serial skin assessment. Routine preoperative investigations and pre-anesthetic evaluation were completed, with prophylactic parenteral antibiotics administered one hour before surgery. All surgical procedures were performed under spinal or combined spinal-epidural anesthesia with patients positioned supine. Surgical postoperatively, antibiotic prophylaxis continued for three days intravenously followed by ten days orally. Immediate postoperative radiographs confirmed adequate reduction and fixation. Non-weight- bearing ambulation and static quadriceps exercises were initiated on the first postoperative day. Following suture removal at 14-15 days, patients progressed to active knee range-of-motion exercises. Complete non-weight-bearing status was maintained for six weeks postoperatively. Patients underwent systematic follow-up at two weeks, six weeks, three months and six months postoperatively, continuing until complete bony union and maximal functional recovery. Clinical union was defined as fracture site stability with absence of abnormal mobility, while radiographic union required bone trabeculae bridging in three of four cortices. Functional outcome assessment utilized the Knee injury and Osteoarthritis Outcome Score (KOOS), comprising 42 items organized into five independently scored subscales: Pain (9 items), Symptoms (7 items), Activities of Daily Living (17 items), Sport and Recreation Function (5 items) and knee-related Quality of Life (4 items). Each item employs a five-point Likert scale, with subscale scores transformed to a standardized 0-100 scale where 0 represents extreme dysfunction and 100 indicates no problems. The KOOS demonstrates robust psychometric properties including high test-retest reliability (ICC 0.75-0.97) and superior responsiveness for tibial plateau fractures. Patient data were systematically retrieved from medical records after obtaining consent, with all information recorded on standardized case report forms.

## Results and Discussion:

The study included a total of 30 patients with a mean age of 44.33 ± 13.19 years (range: 18-65 years). The majority of patients (86.6%) fell within the 21-60 year age bracket, representing the working-age population. The cohort showed a predominant male population (76.7%, n=23) and a slight predominance of left-side injuries (53.3%, n=16) (Table 1 - see PDF). This demographic profile is consistent with established literature on tibial plateau fractures. Vadadoriya *et al.* [14] reported a similar mean age of 42.5 years and a 68% male predominance in their prospective study. The observed age distribution reflects the typical pattern of high-energy trauma affecting the active, working-age population, as originally documented by Schatzker *et al.* in their seminal classification study [15]. Regarding clinical presentation, 46.7% of patients were admitted within 5 days of injury, with a mean admission time of 7.43 ± 4.72 days. While 73.3% of the cohort had no comorbidities, Diabetes Mellitus Type-2 was present in 23.3% of cases (Table 2 - see PDF). The delay in presentation observed in this study (mean 7.43 days) is comparable to findings by Stevens *et al.* [16], who reported a mean delay of 6.8 days in resource-limited settings. However, this contrasts with general recommendations for early intervention within 48-72 hours to optimize outcomes, as emphasized by Honkonen [17]. Surgical management was evenly distributed, with 50% of patients undergoing Open Reduction and Internal Fixation (ORIF) with dual plating and 50% undergoing ORIF with single plating. Notably, no postoperative complications were recorded in this series. This absence of complications compares favorably with infection rates of 5-15% and hardware-related complications of 8-12% commonly reported in larger series. The excellent safety profile may be attributed to appropriate surgical technique selection, adequate rehabilitation protocols and careful patient selection criteria. Fracture classification analysis revealed a predominance of complex injury patterns. Schatzker Type-5 fractures were the most common (46.7%), followed by Type-6 (30%), indicating a high incidence of complex bicondylar fractures. This distribution differs significantly from Western studies, where Type-1 and Type-2 fractures are typically more prevalent. (Table 3 - see PDF) However, it aligns with patterns reported in developing countries where high-energy trauma mechanisms predominate. Girotra *et al.* [18] documented a similar distribution in an Indian population study, noting 51% Type-5 and 28% Type-6 fractures. The predominance of complex fracture patterns in our cohort reflects the high-energy nature of the injuries, consistent with findings by Barei *et al.* [19], who reported increased complexity in motorcycle and vehicular trauma cases (Table 4 - see PDF). The majority of patients achieved union between 14 and 15 weeks (63.4% cumulative).These findings are consistent with literature reports for complex tibial plateau fractures. Mallik *et al.* [20] reported a mean union time of 14.8 weeks for bicondylar fractures treated with internal fixation. The slightly prolonged union time compared to simpler fracture patterns (typically 12-14 weeks) reflects the complexity of the cases included in this series, as documented by Papagelopoulos *et al.* [21] in their comprehensive review. Functional outcomes demonstrated significant progressive improvement across all Knee Injury and Osteoarthritis Outcome Score (KOOS) domains from 6 weeks to 6 months (Table 5 - see PDF). These outcomes are superior to those reported by Zhu *et al.* [22]. The pattern of progressive improvement mirrors findings by Gálvez-Sirvent *et al.* [6], who demonstrated continued functional gains up to 12 months post-operatively, with the most significant trajectory occurring in the first 6 months.

## Conclusion:

We show that despite a predominance of complex bicondylar fractures (76.7%), all patients achieved successful bone union at a mean of 15.03 weeks without complications following open reduction and internal fixation. This clinical success was mirrored by excellent functional recovery, evidenced by statistically significant improvements in overall KOOS scores from 51.80 to 85.73 over six months (p<0.001), thereby validating the KOOS as a reliable and responsive measure for assessing postoperative.
